# Breathing maneuvers as a metabolic coronary vasodilator for first-pass perfusion MR imaging

**DOI:** 10.1186/1532-429X-17-S1-Q115

**Published:** 2015-02-03

**Authors:** Tiago Teixeira, Gobinath Nadeshalingam, François Marcotte, Matthias G Friedrich

**Affiliations:** 1 Montreal Heart Institute, Montreal, QC, Canada; 2 Université de Montreal, Montreal, QC, Canada

## Background

CMR can detect myocardial ischemia by first-pass perfusion and by oxygenation-sensitive CMR (OS-CMR) imaging. While the former can reliably determine myocardial blood flow, the latter integrates other determinants of myocardial oxygenation. Simple breathing maneuvers can trigger a coronary vascular response, which can be monitored by OS-CMR imaging.

## Methods

We studied 24 healthy volunteers (37 ± 12 years; 62.5% men) in a clinical 3T MRI system. Each exam included three sets of first pass perfusion images, (1) at rest and, after 1 minute of hyperventilation during (2) a short breath-hold (SBH) or (3) a long breath-hold (LBH), performed in random order. A reader blinded to the maneuver applied, analyzed signal intensity upslope, upslope index and time between 20 and 80% of maximal signal. For inter-observer variability, a different, blinded, reader repeated the analysis in 4 volunteers.

## Results

**Figure 1:**
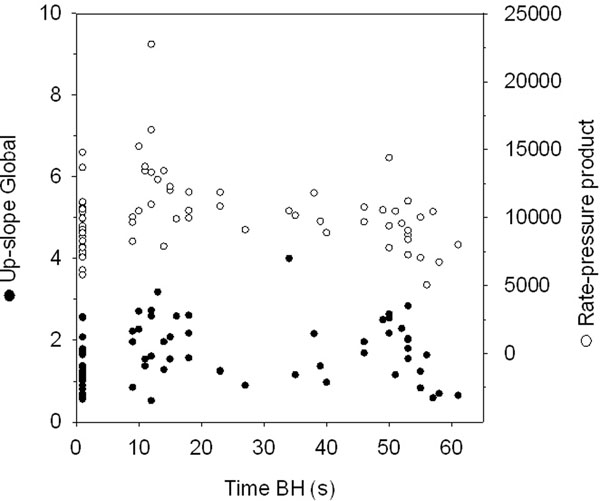
Individual values of upslope and rate-pressure products trough time of breath-hold. The upslope curve (filled circles) shows two peaks.

Demographics and LV function data are presented in Table 1. All volunteers tolerated the breathing maneuvers well and completed the study protocol. The average upslope at rest was 1.34 ± 0.58, and increased by 39% during the SBH (1.86 ± 0.70; p < 0.05), diminishing to 1.77 ± 0.82 at the LBH step. The upslope started at 13.8 ± 5.5 and 49.5 ± 7.3 seconds of breath-hold, respectively, on SBH and LBH. Figure 1 shows the relationship between time of breath-hold after hyperventilation and both the individual values of up-slopes and rate-pressure products (RPP). The upslope curve shows two peaks, a early one (15 seconds) coinciding with the peak of the RPP curve; a second one at about 50 seconds, not promoted by the RPP. The upslope index, which accounts for the arterial input, was higher at this late step (rest: 0.077 ± 0.016; SBH: 0.083 ± 0.015; LBH: 0.095 ± 0.019; p < 0.01), as was the myocardial perfusion reserve index (1.25 ± 0.22 vs. 1.09 ± 0.17). In a multiple regression model that included gender, RPP, breath-hold time, caffeine intake, BSA-indexed mass and set order, only gender, RPP and breath-hold time were independently and significantly related to the upslope (R= 0.771; p < 0.001). A different reader repeated the analysis in 4 volunteers; the intra-class correlation for the up-slope was excellent, of 0.990 (95% CI: 0.943-0.997; p < 0.001).

**Table 1:** Sample demographics and left ventricular function

Volunteer characteristics	All N=24	Men N=15(62.5%)	Women N=9(37.5%)	p-value
Age (years)	37 ± 12	41 ± 14	32 ± 7	ns

Height (cm)	171 ± 7	175 ± 6	165 ± 5	< 0.001

Weight (Kg)	69 ± 10	74 ± 7	62 ± 9	< 0.01

SBP (mmHg)	128 ± 15	134 ± 13	116 ± 9	< 0.01

DBP (mmHg)	74 ± 8	75 ± 8	73 ± 8	ns

HR (bpm)	68 ± 10	64 ± 9	75 ± 9	< 0.01

Caffeine intake [n(%)]	14 (58.3	10 (41.7)	4 (16.7)	ns

Routine Exercise [n(%)]	17 (70.8)	10 (41.7)	7 (29.2)	ns

CMR findings				

EDV/BSA (ml/m2)	91.1 ± 14.8	96.1 ± 16.1	82.8 ± 7.3	< 0.05

ESV/BSA (ml/m2)	37.1 ± 7.6	39.9 ± 8.0	32.5 ± 3.9	< 0.05

MM/BSA (g/m2)	57.0 ± 14.0	64.8 ± 11.3	44.0 ± 6.2	< 0.001

EF (%)	59.4 ± 4.0	58.6 ± 4.5	60.7 ± 2.7	ns

SBP - systolic blood pressure; DBP - diastolic blood pressure; HR - heart rate; EDV - end-diastolic volume; ESV - end-systolic volume; MM - myocardial mass; BSA - body surface area; EF - ejection fraction.

## Conclusions

The blood flow response to simple breathing maneuvers can be demonstrated by first-pass perfusion CMR, with a early peak dependent on RPP increase, and a late peak due to the vasodilatory effect of long breath holds. Confounding effects of breathing may also have implications for CMR first-pass perfusion imaging performed with pharmacological vasodilators.

## Funding

None.

